# Temporal Context affects interval timing at the perceptual level

**DOI:** 10.1038/s41598-020-65609-6

**Published:** 2020-05-29

**Authors:** Eckart Zimmermann, Guido Marco Cicchini

**Affiliations:** 1 Institute for Experimental Psychology, Heinrich Heine University Düsseldorf, Universitätsstraße 1, 40225 Düsseldorf, Germany; 2 Institute of Neuroscience, CNR, Via Moruzzi, 1, 56124 Pisa, Italy

**Keywords:** Sensory processing, Visual system

## Abstract

There is now ample evidence that when observers are asked to estimate features of an object they take into account recent stimulation history and blend the current sensory evidence with the recent stimulus intensity according to their reliability. Most of this evidence has been obtained via estimation or production paradigms both of which entail a conspicuous post-perceptual decision stage. So it is an unsolved question, as to whether the trace of previous stimulation contributes at the decision stage or as early as the perceptual stage. To this aim we focused on duration judgments, which typically exhibit strong central tendency effects and asked a duration comparison between two intervals, one of which characterized by high uncertainty. We found that the perceived duration of this interval regressed toward the average duration, demonstrating a genuine perceptual bias. Regression did not transfer between the visual and the auditory modality, indicating it is modality specific, but generalized across passively observed and actively produced intervals. These findings suggest that temporal central tendency effects modulate how long an interval appears to us and that integration of current sensory evidence can occur as early as in the sensory systems.

## Introduction

Our sensory systems continuously inform us about the color of objects, the identity of faces, the duration of events. Some of these judgments are reached with good sensory information but many, being it for short exposure, lack of attention or scarce sensory information are based on far-from-ideal sensory information. There is now growing evidence that in such conditions observers estimate or reproduce stimulus qualities with a strong central tendency which steers responses towards the average of previous stimulation history^1,2^.

One of the domains where these effects are particularly strong is time perception, in which judgements can gravitate towards an intermediate interval (Vierord’s Law^3–7^. Despite the pressing need for accurate temporal estimations, judgments do not have Weber Fractions as low as other sensory systems^8,9^ and are susceptible to many factors such as action, intention and execution^10–12^, attention^13^, pace of presentation^14,15^ and masking^16^. In line with this, central tendencies in duration reproduction tasks can be quite powerful and distort estimates up to 60%^8^. For this reason, temporal judgments have proven as a useful benchmark to demonstrate many properties of central tendencies. In particular it has been shown that reliability of each stimulus is paramount in determining central tendency^2,8,17–20^ and that regression towards the mean is an effective strategy to tame the response errors in the face of noisy sensory signals^2,18,21^.

Importantly, much of the literature on central tendency effects is built upon estimation or reproduction paradigms which leave ample room for post-perceptual decision mechanisms^20^. However, it is unclear whether similar strategies are applied as early as in the perceptual systems. Indeed, in a seminal experiment Roach and colleagues^21^ interleaved stimuli belonging to distinct ranges and distinct modalities, and asked either to perform temporal reproduction with the same motor act or with two distinct motor acts. The authors found that when one motor output is requested, responses converge towards a common average; separate central tendencies emerged only if subjects were asked to perform distinct motor acts for the two modalities indicating a crucial role of the motor planning.

Given the usefulness of central tendencies in reducing error and the fact that other perceptual judgments can be affected by recent stimulus history^22–24^ it would be rather surprising if mechanisms for duration perception were immune from such a mechanisms. The evidence in this respect is quite scarce and fragmented. On one hand, in the same manuscript Roach *et al*. have reported that after some practice (4 or more sessions of 140 trials each) separate priors emerge even if a single motor response was requested. This indicates that sensory systems have a capability of storing temporal context, but it requires time. On the other hand a couple of recent works have shown that when stimuli follow a regular pattern, the perceived timing of the last stimulus of the sequence is biased towards the putative time belonging to the rhythm, indicating some capacity to warp perceived time to comply with more wholistic representations^14,15^. Yet, to date, a clear demonstration of central tendency in temporal perceptual judgments is still missing. The central research question of the present study asks if temporal regression of the mean exists on the perceptual level.

Measuring perceptual distortions induced by the temporal context is challenged by the fact that if a distortion of perceptual time were taking place, it would affect equally all stimuli that subjects have to compare and null effect would result. Our method to overcome this difficulty leverages on the fact that intervals carrying high uncertainty should display stronger context effects than intervals with better temporal resolution. So a perceptual comparison between a high uncertainty and a low uncertainty stimulus should reveal if context effects are taking place^25^. In this work we exploited this principle and introduced high uncertainty in one of the two stimuli either masking the visual stimulus, or presenting the two auditory markers to the two ears separately. We demonstrate that this simple manipulation uncovers strong context effects in a purely perceptual comparison task. Further we show that temporal context effects are modality specific and they can be strong enough to override other temporal distortions like those introduced by voluntary action.

## Results

### Experiment 1: Baseline and visual distortion

**Figure 1:**
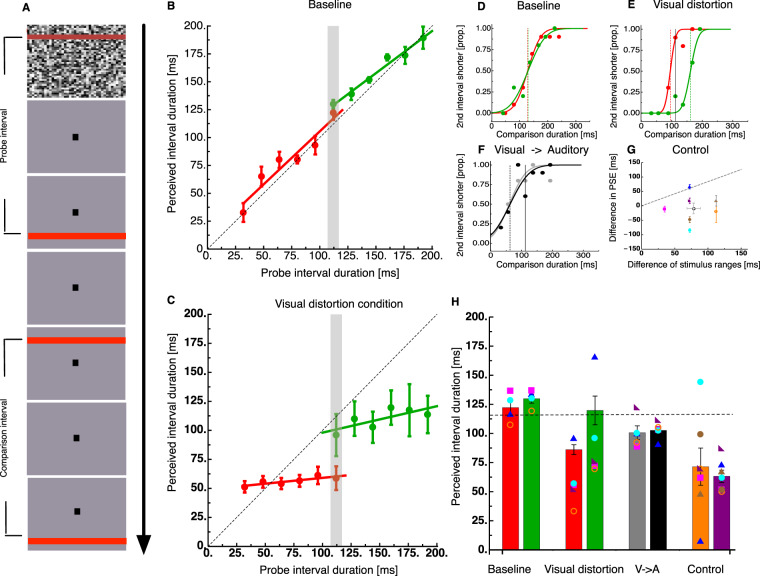
Temporal context affects perception of time. (**A**) Stimulus sequence in the visual illusion condition. A probe interval of fixed duration has to be compared with a comparison interval whose duration varied and allowed to extract psychometric functions. In the baseline condition both the probe and the comparison intervals were delimited by a red bar upon an uniform background, in the visual illusion condition (displayed here) the fixed probe interval was delimited by a bar over a luminance mask and a bar upon an uniform background. Such manipulation is known to introduce temporal distortions and/or increase in uncertainty (Zimmermann *et al*., 2014; 2016). (**B**) Perceived duration of stimuli in the baseline condition either in a session comprised only of short intervals (33–117 ms -red curve) or long intervals (117–200 ms - green curve). Isolated data points indicate perceived duration of each probe interval along with standard errors of the sample mean. Grey region delimits the 117 ms stimulus which has been presented in both temporal context conditions (short and long intervals). (**C**) Data from the “visual illusion condition” in which one of the two bars of the probe was presented superimposed on a mask. (**D,E**) Psychometric functions of a representative subject judging the duration of a 117 ms stimulus; red data points indicate data when extracted from the “brief intervals” condition, green data points are extracted from the “long intervals” condition. Psychometric curves indicate best fitting cumulative gaussian functions which estimate the point of subjective equality at which probe and comparison are perceived as having the same duration (indicated by vertical lines). (**F**) Psychometric curves for the visual-to-auditory transfer condition in which presentation of visual intervals provided a temporal context (86% of trials) and interspersed auditory intervals of 117 ms measured the transfer of the context to other modalities. As before the range of intervals could be short (33–117 ms - grey) or long (117–200 - black). Psychometric curves display only the crucial visual trials of 117 ms where transfer was measured. (**G**) Control condition. In order to control for the susceptibility of judgments to the durations of the second comparison interval we asked a comparison of a 117 ms visual stimulus with a mask either using a range of comparisons extracted from a uniform distribution either of brief or long intervals. The effect on perceived time when testing with the two temporal contexts is plotted against the average difference oft he probe intervals in the two sessions. Symbols indicate individual data, hollow symbol average effect. The thin dashed line indicates a prediction of a model which ascribes the whole central tendency  to the difference in the probe intervals used in the two sessions. (**H**) Average perceived duration in the four experimental conditions shown in panels D-G. Error bars indicate the standard error of the sample mean. Colored symbols show individual subject data. The dashed line shows veridical interval duration (117 ms).

We measured how time perception is affected by stimulus range asking subjects to compare a probe and a comparison interval (see Fig. 1A). Both intervals were purely visual, defined by the brief presentation of two visual bars. In separate sessions, probe interval durations ranged from 33 to 117 ms or from 117 to 200 ms. The comparison interval that followed the probe varied in duration served to derive the Point of Subjective Equality (PSE). We first ran the experiment in a baseline condition in which probe and comparison intervals were identical stimuli except for their duration. Figure 1B shows perceived duration of the probe interval in the baseline condition averaged across all observers. We estimated the regression lines for intervals with low duration (red color, regression: 10.53 + 0.95×) and for intervals with high duration (green color, regression: 44.48 + 0.75×). The size of the range effect can be quantified by the slope of the regression line. If the results are unaffected by a range effect, all data should lie on the identity line and the regression slope would be 1. Any shift of the slope towards 0 would indicate the strength of a regression to the mean. In order to test the putative range effect statistically, we calculated regression lines within each observer and tested the respective slopes of all observers against 1. In the baseline condition we found that slopes were not statistically different from 1 for low intervals (t(3) = −0.57, p = 0.31) but only for high intervals (t(3) = −3.53, p = 0.02). See supplementary material for individual data. When we ran the experiment in the visual distortion condition, the first visual bar in the probe interval was presented on top of a whole-field mask (see Fig. 1A). Earlier studies have shown that this condition leads to an increase of discrimination thresholds along with a compression of apparent time^16,26^. Figure 1C shows perceived duration of the probe interval in the distortion condition averaged across all observers from Experiment 1. In contrast to the baseline condition when the probe interval contained a mask, estimates gravitate heavily towards the average interval of the session (average slope for low intervals = 49.04 + 0.10×, average slope for high intervals = 75.24 + 0.22×). T-tests confirmed that slopes for low (t(4) = −6.2, p < 0.005) and high (t(4) = −9.9, p < 0.001) intervals were significantly shallower than 1, indicating a genuine regression towards the average, even in a perceptual task.

### No transfer of visual temporal context to audition

**Figure 2:**
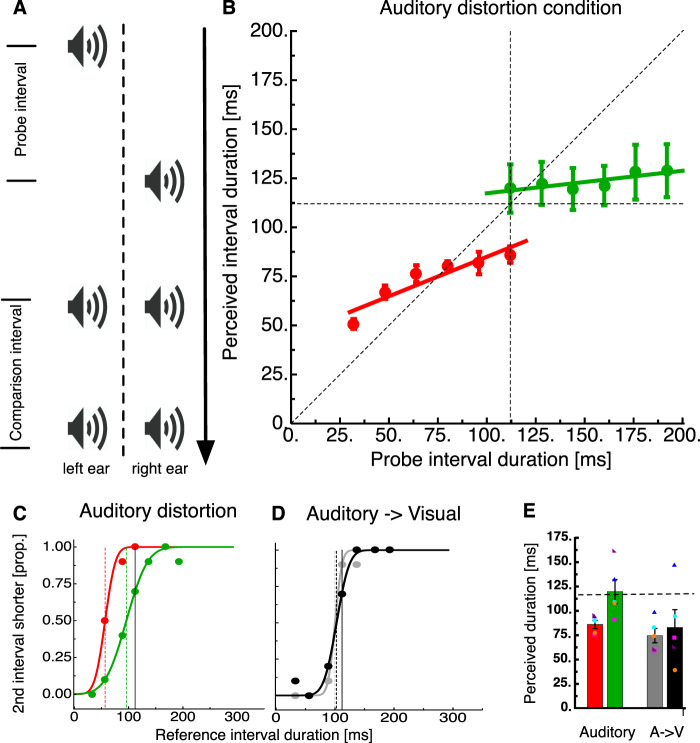
Temporal context does not generalize across modalities. (**A**) Stimulus timeline for an auditory version of the experiment in which the probe intervals was marked by a monaural presentation of a beep, and comparison interval by binaural presentation. These trials constituted 6/7 of trials and provided a temporal context. The remaining  1/7 of trials comprised a visual stimulus similar to that of the visual illusion condition. (**B**) Perceived interval duration of an auditory stimulus either embedded in a brief interval context or in a long interval context. Conventions are similar to Fig. 1B and display that also in the auditory modality temporal intervals can be attracted towards the center of the stimulus distribution. (**C**) Psychometric curves for 117 ms auditory probe intervals either in a brief interval context (red) or in a long interval context (green). Dashed lines indicate PSE, all other conventions are like in Fig. 1D–G. (**D**) Psychomeric curves for a 117 ms visual interval in the transfer trials presented amongst either brief or long auditory intervals. (**E**) Summary statistics for the conditions of Fig. 2C,D, error bars represent the standard error of the sample mean. Colored symbols show individual subject data. The dashed line shows veridical interval duration (117 ms).

Next, we asked about transfer of central tendency effects between the visual and the auditory modality. To this end, we adopted and modified an auditory task that has been demonstrated to induce a temporal distortion^10^ (see Fig. 2A). The distortion modifies duration perception if temporal interval markers are presented to different ears compared to when they are presented to the same ears. We therefore presented an interval start marker to one ear and after the duration of the interval, the second marker to the other ear. Observers had to compare the duration of this interval to a comparison interval in which interval start and end markers were presented to both ears. Although for this illusion only a reduction of perceived duration had been reported, we found in piloting experiments that this illusion was also amenable for regression to the mean. We interspersed these auditory trials between the visual trials such that trials containing the auditory distortion represented only a fraction (1/7) of all trials. The physical duration of the auditory distortion in this experiment was always 117 ms whereas the visual trials could be either short (33 to 117 ms) or long (117 to 200 ms). Modulation of duration estimation by interval context was indicated by differences in judgements of the 117 ms interval when presented together with shorter or longer visual intervals (see Fig. 1D–F for example psychometric functions). On average, interval duration judgements for the visual illusion were strongly biased towards the mean (see Fig. 1H). However, in the randomly interleaved transfer trials containing the auditory stimuli, there was no modulation by interval context and estimations were virtually identical (see Fig. 1F,H). Transfer of central tendency effects between the visual and the acoustic modality was also absent in the average data as can be seen in Fig. 1H. As we collected these data along with the complementary experiment where the transfer was from audition to vision (Experiment 2), we decided to analyze the entire dataset together so for statistical analysis please refer to the section on Experiment 2.

### Subjects perform a genuine comparison between the stimuli

Since the probe interval is noisier than the comparison due to the presentation of the mask, subjects could choose to base their judgement on the average duration of all comparison intervals^27^. In this regime, they would ignore the probe interval and respond whether the current comparison interval is shorter or longer than the average of all previous comparison intervals. To measure how strong this effect was in our dataset we repeated the visual distortion condition considering only one duration for the probe interval (117 ms). We ran separate blocks using two well separated interval duration ranges for the comparison, i.e. the allegedly more informative, stimulus. If subjects ignored the probe stimulus and based their responses only on the relative duration of the comparison intervals, the two interval duration ranges should yield different psychometric curves gravitating around the average comparison stimulus.

In order to estimate the putative influence of the comparison intervals on the 117 ms probe intervals we calculated the change in PSE introduced by the two different temporal contexts and plotted it against the mean duration difference between the two conditions (long probes – short probes, see Fig. 1G). The colored symbols mark the different subjects. The hollow data point represent the average. It is clear from the graph that the average drift of the psychometric functions even amounts to a negative effect (−9.04 ± 18.61). Besides the average values, also the correlation between these two variables is weak (r = 0.08) and likely reflects a non-dependence between the two measures (BF = 0.46).

To better frame this result we also display the prediction of a model which ascribed the whole central tendency to the range of duration of probe intervals. In the visual distortion condition, we found slopes of 0.10 and 0.22 in the short interval and long interval conditions corresponding to a weight of the context (1-slope) of 90% and 78% respectively (on average 84%). For this reason if all of the effect reported in that experiment was due to the diversity between the probe intervals we should have found that PSEs in the two probe conditions shift about 84% of the difference of the probe ranges (dashed line y = 0.84×). Comparison of this model to the data yields the impression of a rather poor fit. This is substantiated when calculating the maximum likelihood that such a model explains the data and dividing it by the likelihood that the best fitting linear model produces the data. The ratio between the two likelihoods is 105.8, indicating that overall this hypothesis is capturing very little of the data.

### Experiment 2: auditory distortion

Next, we tested presence and transfer of central tendency effects in the auditory modality. In order to lower the reliability of auditory signals we presented one auditory interval marker to the left and the other to the right ear (see Fig. 2A). As can be seen in Fig. 2B, interval judgments were strongly modulated by central tendency effects under this condition. In order to test the putative transfer of auditory regression to visual perception, we interspersed trials containing the visual distortion between the auditory trials.

The participant shown in Fig. 2C showed a clear central tendency effect for auditory duration judgements with PSEs changing even twofold. Average data (Fig. 2B) revealed a strong group effect (slope for low intervals = 44.74 + 0.40×, slope for high intervals = 105.85 + 0.12×). A t-test confirmed that slopes for low (t(4)=−6.19, p < 0.001) and high intervals (t(4) = −9.94, p < 0.001) were significantly shallower than 1. However, in the transfer trials their judgements did not differ between trials that were embedded in shorter or in longer regression trials (see Fig. 2D). What can be seen for both transfer conditions (gray and black) is an underestimation of the 117 ms interval which reflects time compression induced by the presentation of the mask in the probe interval. This underestimation is also reflected in the average data (see Fig. 2E). On average, regression to the mean of auditory judgements but no transfer onto the visual modality occurred (see Fig. 2E). A 2 × 2 × 2 repeated measures ANOVA with the factors Transfer direction (visual->auditory/auditory->visual), Trial Type (regression trial/transfer trial) and Interval Context (short/long) revealed a significant main effect for the factor Interval Context (F(1,4) = 22.08, p < 0.01) and a significant interaction effect between the factors Trial Type and Interval Context (F(1,4) = 10.75, p = 0.03).

## **Experiment 3: Active**/**passive transfer**

**Figure 3:**
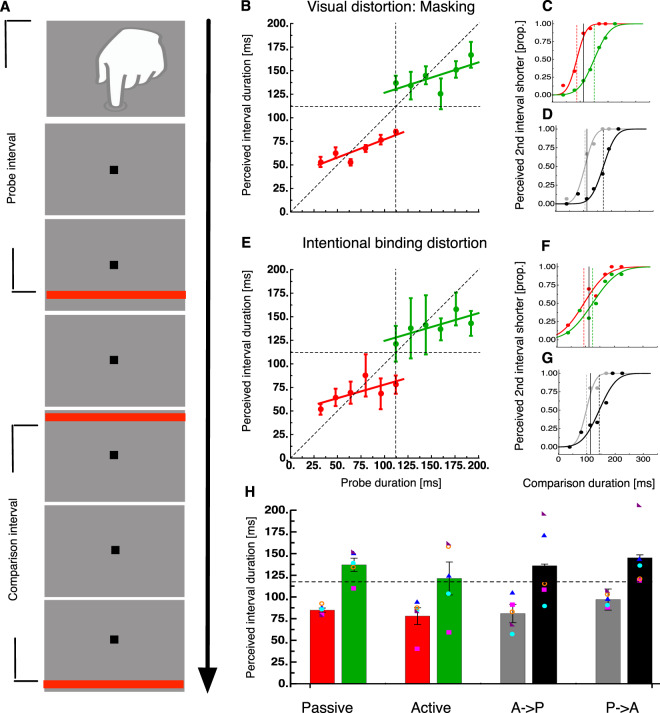
Temporal context generalizes across motor actions. (**A**) To test the generality of the temporal context effect, we introduced a further condition in which the interval could be actively produced. In this case the probe interval was delimited by a key-press performed by the observer and a visual stimulus presented after a given interval. Such “active trials” could either be the majority during the session (and the few passive trials would be transfer trials) or could themselves be interspersed in between passive trials (in which case they are active-transfer trials). (**B**) Perceived duration of active trials in a session where active trials are the majority of the trials. Conventions are the same as in Fig. 1B. Data indicate that temporal context takes place also between actively produced intervals. (**C,D**) Sample psychometric curves either for 117 ms active trials or 117 ms passive trials. Temporal context was always comprised of active trials either belonging to a distribution of brief stimulus (green or grey) or long stimuli (red or black). The curves separate depending upon the range of durations employed in the context. (**E–G**) Similar plots as **(B–D**). The temporal context is provided by passively produced trials. Red and green colors again refer to context trials (in this case, passive visual trials alike those of Experiment 1) and gray-black  colors refer to transfer trials (in this case active trials). Error bars represent the standard error of the sample mean. (**H**) Summary statistics for the conditions of Fig. 3C,D,F,G, error bars represent the standard error of the sample mean. Colored symbols show individual subject data. The dashed line shows veridical interval duration (117 ms).

We also asked about transfer of central tendency effects between passively observed and actively produced distortion of time. We chose the intentional temporal binding effect where a button press leads to a temporal interval compression between the press and the following sensory event^11^ (see Fig. 3A). We first tested interval judgements for the passive mask illusion again. As in Experiment 1, we found regression to the mean for both interval ranges (see Fig. 3B, slope for low intervals = 38.63 + 0.38 ×, slope for high intervals = 94.28 + 0.32 ×). A t-test confirmed that slopes for low (t(4) = −9.18, p < 0.001) and high intervals (t(4) = −13.33, p < 0.001) were significantly shallower than 1.

In order to measure the transfer of the regression effect, we interspersed trials between the passive trials that contained the active intentional temporal binding paradigm (see Fig. 3A). As in Experiment 2 these trials represented 1/7 of all trials in the session. In trials containing the active illusion the probe interval start was marked by a button press performed by the subject and the end by a flashed visual bar. The duration of these intervals had to be compared against intervals defined by two bar flashes.

Temporal judgements for the passive and for the active 117 ms intervals are shown in Fig. 3C,F for one subject. In both distortions, this subject demonstrated a clear central tendency effect. We then tested interval judgements for the active distortion. Similarly to the passive distortion we found regression to the mean (see Fig. 3E, slope for low intervals = 49.14 + 0.29×, slope for high intervals = 95.03 + 0.29 ×). A t-test confirmed that slopes low (t(4) = −8.14, p < 0.001) and high intervals (t(4) = −2.9, p < 0.05) were significantly shallower than 1.

These regression effects were found for the active distortion but also for the interspersed passive distortion (see Fig. 3D,G for a single subject). On average, regression transferred almost completely between the passive and active distortions (see Fig. 3H). A 2 × 2 × 2 ANOVA with the factors Transfer direction (Passive->Active/Active->Passive), Trial Type (Regression trial/Transfer trial) and interval context (short/long) revealed a significant main effect for the factor interval context (F(1,4) = 14.46, p = 0.019). Thus, both regression and transfer trials were affected by the interval context. There was no statistical evidence for a difference between transfer direction or trial types.

## Discussion

In this study we investigated temporal duration judgments and showed that even perception is affected by central tendency. Principally, it is impossible to measure central tendency when two stimuli have equal sensory resolution. However, when we introduced additional noise into one of the intervals by presenting a mask, or by playing sounds to both ears separately or by active button pressing, we found that duration matches were attracted towards the mean of all presented intervals. Further, perceptual regression towards the average temporal duration was modality-specific. We did not observe any transfer of regression between visually and auditory defined intervals. However, within the visual modality regression to the mean did transfer between passively observed and intervals where one of the two intervals was delimited by an action. These findings are consistent with the idea of central tendency effects taking place at the perceptual level.

We found that if the setup allows distortions and central tendency effects to emerge - i.e. when intervals are presented in a range of durations - strong regression effects occur and can even cancel out the distortion. This has been overlooked in the previous literature as temporal distortions were mostly tested with a single interval duration. The intentional binding effect - that was used also in the present study - for instance, usually is investigated with 100 ms interval, resulting in temporal compression^28^. As we show here, regression to the mean becomes the dominant effect when a range of intervals is tested. Whether we found time compression or expansion for our 117 ms interval depended on the range of intervals in which it was embedded. Thus, conclusions drawn from investigations into temporal distortions can be generalized only to regular presentations of identical interval durations. As soon as interval durations vary - as in real life - central tendency effects take the lead.

Our results are clearly in line with a Bayesian explanation of central tendency effects^2,8,29^ as the interval which bear more uncertainty is the one that undergoes the strongest pull towards the central interval. In this respect it would have been interesting to fit the data with a Bayesian model. However applying any flavor of the aforementioned models presents several challenges. In a first place, it is difficult to extract sensory resolution levels from an experiment like ours because perceptual estimates undergo a contextual effect and, in these conditions, the measurable noise reflects mostly the noise of the prior, not that of the probe stimulus. Further one cannot assume that the high reliability comparison stimulus is immune from the prior. This implies that also the noise of the representation of the comparison stimulus is determined by the interaction with the prior. This is catastrophic because if the comparison stimulus is compressed this impacts both on the measured regression effect in the 2IFC (decrease) both the measured JND of the 2IFC (increase). One possibility would be to leverage on the fact that context effects do not transfer across modalities and measure JNDs in transfer trials. Indeed when we did so, we found that both of our manipulations (masking one bar in vision, presentation to separate ears in audition) yielded unusually high noise levels for each modality (Weber Fractions of 36 ± 9% and 17 ± 2% respectively). In theory it is possible to repeat these measures for all durations and for all types of stimuli (of low and high reliability). However given that this data collection is highly inefficient (because it employs only the transfer trials which are only 14% of the trials) we would leave it for a future study.

Interestingly in our paradigm we have measured a regression effect also for the auditory modality which in other circumstances has been found immune to regression effects^8^. However it is worth of notice that our stimuli bear some differences with other typical setups. Firstly our auditory markers are rather long (about 250 ms) and it has been shown that markers beyond 200 ms yield worse JNDs^30^. Secondly the two markers overlap in time, therefore the overall power modulation in time is less that an interval marked by brief clicks and with a distinct silence period in between. Not least, binaural presentation, may have also contributed as it has been demonstrated already that binaural sounds are more prone to perceptual illusions^10^. We speculate therefore that both the perceptual distortions and the regression may be higher because of a higher uncertainty associated with these kind of presentations.

As a by-product our research revealed that prior information is rather specific for the stimulus employed, consistent with sensory-specific effects in time perception^31^. This was directly tested in Experiment 2 which showed that temporal context of one modality did not affect temporal judgments of the other modality. At the same time, quite unexpectedly, we found a similar lack of transfer also in the control experiment (see Fig. 1G). In this paradigm we tested a comparison between low reliability visual stimuli and high reliability visual stimuli. If a common prior for visual stimuli existed, the mere presentation of the comparison stimuli should have yielded an attractive effect of the high reliability stimuli upon the low reliability ones. However this did not happen, suggesting the possibility that even within the same modality multiple traces of sensory history could co-exist and they may be tagged to the event and the stimulus that marks it. This is not in contrast with optimal integration theory as stimulus similarity is a key variable in enabling context effects^19,32,33^.

Our results may seem at odds with a recent report which has argued that central tendency in temporal reproduction leverages on a supramodal prior occurring in motor decision stages^21^. However, there are several cautionary notes before the two results are contrasted. Firstly, the two experiments tap on rather distinct duration ranges. Our stimuli are very brief, (typically 117 ms and maximum 200 ms); Roach’s stimuli ranged from 200 ms to 2 seconds.

According to a large body of literature perception of time between sub-second and supra-second intervals is subserved by different mechanisms^8,34–36^. Further there is good evidence that intervals below one second are processed independently by each modality, however stimuli above one second are processed by a common timing mechanism^34,37^. This could explain in part why we do not find transfer between modalities whereas Roach *et al*. (2017) have some conditions in which there is transfer between modalities.

Our findings are also related to the growing literature on the attractive effect exerted by a preceding stimulus, known as serial dependence^18,38,39^. Similarly to central tendency, also serial dependence is attractive and obeys laws of optimal perception^14,30^. Also the field of serial dependence is under a vibrant debate concerning whether its origins lie in perception, perceptual judgments or motor response^33,40–42^. One of the emerging results in the serial dependence literature is that under circumstances predicted by lawful Bayesian integration, also a tangible perceptual component can be documented^43^ which bears a strong similarity to what reported here and reiterates the pivotal role of sensory reliability in contextual effects.

In conclusion, our data suggest that central tendency effects in temporal interval estimations occur at the perceptual level. In particular it suggests that sensory systems compute and make use of sensory statistics over the last few minutes, a peculiar function which should be supported by a dedicated, neural mechanism and shapes the way objects appear to us.

## Materials and methods

### Participants

Four subjects (one male author and one male and two female naive subjects; mean age: 28 years) participated in the baseline condition of Experiment 1. Five different subjects (one male author and one male and three female naive subjects; mean age: 27 years) participated in the visual condition of Experiment 1 and Experiment 2. Five different subjects in Experiment 3 (one male author and one male and three female naive subjects; mean age: 27 years). All subjects had normal or corrected-to-normal vision. Subjects gave informed consent. The experiments were carried out along the principles laid down in the Declaration of Helsinki. All experiments were approved by the local ethics committee of the psychological department of the Heinrich-Heine University Düsseldorf.

### Sample Size

In order to plan the experiment we ran an a priori power analysis simulating the experimental paradigm which is focused on measuring the central tendency effect. To this aim, we mirrored our experimental paradigm and asked how many subjects would ensure that a given effect would be detected in 90% of the cases (i.e. power of 0.9). To do so we assumed that each observer would be tested at 7 different duration each requiring a psychometric curves of 60 trials and yielding a PSE. PSEs at each duration would then be calculated. Finally PSE as function of test duration would be plotted and the slope of the fit would indicate the central tendency effect.

In order to provide a reasonable stimulation which incorporated sources of noise we assumed that each judgment would be corrupted by a scalar gaussian noise equivalent to 20% of the duration and also that between subjects regression effects could vary up to 20%.

Even though typical central tendency effects can be very large (up to 50% of the physical duration) we required that our paradigm could be able to detect the presence of an effect of about only 10% (i.e. slopes of 0.9). These simulations (10,000 iterations) suggested that already four subjects would be sufficient to detect such a central tendency in 84% of the cases, increase to 5 subjects would lead to a power of 92%.

### Apparatus

Subjects were seated 57 cm from a CRT monitor (Eizo FlexScan T57S). The visible screen diagonal was 40.5 cm, resulting in a visual field of 40° × 30°. Stimuli were presented on the monitor with a vertical frequency of 120 Hz on a homogeneously gray background (luminance: 11.65 cd/m^2^.

### Procedure

Experiment 1 tested whether visual and auditory temporal intervals would be subject to central tendency effects and whether these would transfer between modalities. In sessions of Experiment 1 interval duration were chosen from a range of 33–117 ms in 6 steps of 16 ms. Experiment 2 tested central tendency effects and transfer between passively observed and actively produced visual intervals. A session of Experiment 1 or 2 contained two trial kinds: Regression and transfer trials. In a session, 6 different probe interval durations of a specific trial kind were tested. Depending on the session this set consisted either of short (33.32–116.62 ms, 6 steps of 16.66 ms) or long intervals (116.62–199.92 ms, 6 steps of 16.62 ms). Interspersed into each session were transfer trials which contained probe intervals that always lasted 117 ms. All trials were presented in randomized order. No feedback about correct estimations was given to participants in any of the experiments reported in this study. In each session of Experiment 1 or 2, a full psychometric function was measured for the 6 regression intervals and the single transfer interval. For each psychometric function 70 trials were measured. To estimate psychometric functions we averaged the 10 responses for each of the 7 comparison intervals within each observer and fitted a cumulative gaussian function to the data.

### Visual intervals: Passively observed

A trial started with the presentation of a fixation point (black, radius: 0.25°, luminance: 0.11 cd/m^2^), which was shown constantly throughout the whole session (see Fig. 1A). After 1000 ms plus a random delay between 0 and 500 ms, a bar stimulus (red, 40° × 2°, luminance: 6.38 cd/m^2^) was presented 10° above screen center. Except in the baseline condition, the bar stimulus was shown on top of a whole screen random-texture mask, consisting of 40 × 30 rectangles (size: 1° × 1°) which each had a randomly assigned luminance on the gray scale level.

Bar and mask were presented for one frame (8.3 ms). The onset of bar and mask marked the start of the probe interval. The duration of the probe interval was randomly selected per trial from a set of 6 possible durations (see Procedure). The end of the interval was marked by another bar stimulus (red, 40° × 2°, luminance: 0.11 cd/m^2^) presented 10° below the screen center for one frame (8.3 ms) without a mask. Thousand ms later, the comparison interval was presented. As for the probe interval, start and end of the comparison interval were marked by a bar stimulus (red, 40° × 2°, luminance: 0.11 cd/m^2^) flashed 10° above and below screen center for one frame (8.3 ms). No mask was presented in the comparison interval. The duration of the comparison interval depended on the probe interval and was randomly chosen from seven equiprobable durations (symmetrically distributed around the probe interval). The following comparison interval durations were selected for the short intervals: For the 33.32 ms interval: (8.33, 16.66, 24.99, 33.32, 41.65, 49.98, 58.31), for the 49.98 ms interval: (16.66, 24.99, 41.65, 49.98, 58.31, 74.97, 83.3), for the 66.64 ms interval: (16.66, 33.32, 49.98, 66.64, 83.3, 99.96, 116.62), for the 83.3 ms interval: (24.99, 41.65, 66.64, 83.3, 99.96, 124.95, 141.61), for the 99.96 ms interval: (24.99, 49.98, 74.97, 99.96, 124.95, 149.94, 174.93) and the 116.62 ms interval: (33.32, 58.31, 91.63, 116.62, 141.61, 174.93, 199.92). The following comparison interval durations were selected for the long intervals: For the 116.62 ms interval: (33.32, 58.31, 91.63, 116.62, 141.61, 174.93, 199.92), for the 133.28 ms interval: (33.32, 66.64, 99.96, 133.28, 166.6, 199.92, 233.24), for the 149.94 ms interval: (41.65, 74.97, 116.62, 149.94, 183.26, 224.91, 258.23), for the 166.6 ms interval: (41.65, 83.3, 124.95, 166.6, 208.25, 249.9, 291.55), for the 183.26 ms interval: (49.98, 91.63, 141.61, 183.26, 224.91, 274.89, 316.54) and the 199.92 ms interval: (49.98, 99.96, 149.94, 199.92, 249.9, 299.88, 349.86).

Subjects had to indicate whether the probe or the comparison interval was shorter (2-IFC task) by pressing the left or right arrow key of the computer keyboard. The subject’s response started the next trial.

### Auditory intervals

Auditory interval markers were delivered via earphones, consisting of a single-pulse (acoustic tom-tom drum sound, ~250 ms duration with a short decay) (see Fig. 1C). In order to define an auditory temporal interval, one sound marking interval start was presented to one ear and another sound marking interval end to the other ear. As for the sessions containing the visual distortion, the duration of the probe intervals was randomly chosen out of 6 possible durations for short and for long intervals (same durations as in Experiment 1). After 1000 ms the comparison interval was presented. In the comparison interval, both, the interval start and end markers were both defined by presenting the sound to both ears. The subject had to indicate which interval appeared shorter by pressing one of the arrow buttons. The response started the next trial.

### Controls

In order to control for the possibility that subjects based their judgements only on the average duration of all comparison intervals instead of the probe intervals, we ran an extra experiment. In two separate sessions, subjects had to estimate the masked 117 ms interval either against comparison intervals that were selected to be within the range of the low interval durations (8.33–199 ms) or against comparison intervals within the range of the high interval durations (8.33–349.86 ms).

### Visual intervals: Intentional temporal binding effect

In actively produced intervals the probe interval start was marked by a button press performed by the subject. Participants were instructed to press the space bar on the computer keyboard and to observe the temporal interval between the button press and the interval end marking stimulus which was a bar (red, 40° × 2°) presented for one frame (8.3 ms) 10° below screen center. As for passively observed intervals, the duration of actively produced probe intervals was randomly chosen out of 6 possible durations (see Procedure). After 1000 ms the comparison interval was presented that was identical to those of the passively observed intervals. When subjects pressed an arrow button to give the response a new trial started. The subject was then free to push the space bar when she or he felt ready.

## Supplementary information

Supplementary Information.

## Data Availability

The datasets generated during and/or analyzed during the current study are available in the Open science framework repository, https://osf.io/8eb27/?view_only=f18de1a4b8d045ae978a69f9f8ede7f1.
