# Supplementary material for: Temporal Context affects interval timing at the perceptual level
Source: Sci Rep. 2020 May 29;10:8767. doi: 10.1038/s41598-020-65609-6 (PMC7260213; doi:10.1038/s41598-020-65609-6)
Supplement: Supplementary file 1 — Supplementary Information. [file 41598_2020_65609_MOESM1_ESM.docx]

Supplementary information to
Temporal Context affects interval timing at the perceptual level

Eckart Zimmermann^1*^ and Guido Marco Cicchini^2

1^ Institute for Experimental Psychology, Heinrich Heine University Düsseldorf, Universitätsstraße 1, 40225
 Düsseldorf, Germany

^2^ Institute of Neuroscience, CNR, Via Moruzzi, 1, 56124, Pisa, Italy


* corresponding author: [eckart.zimmermann@uni-duesseldorf.de](mailto:eckart.Zimmermann@uni-duesseldorf.de)


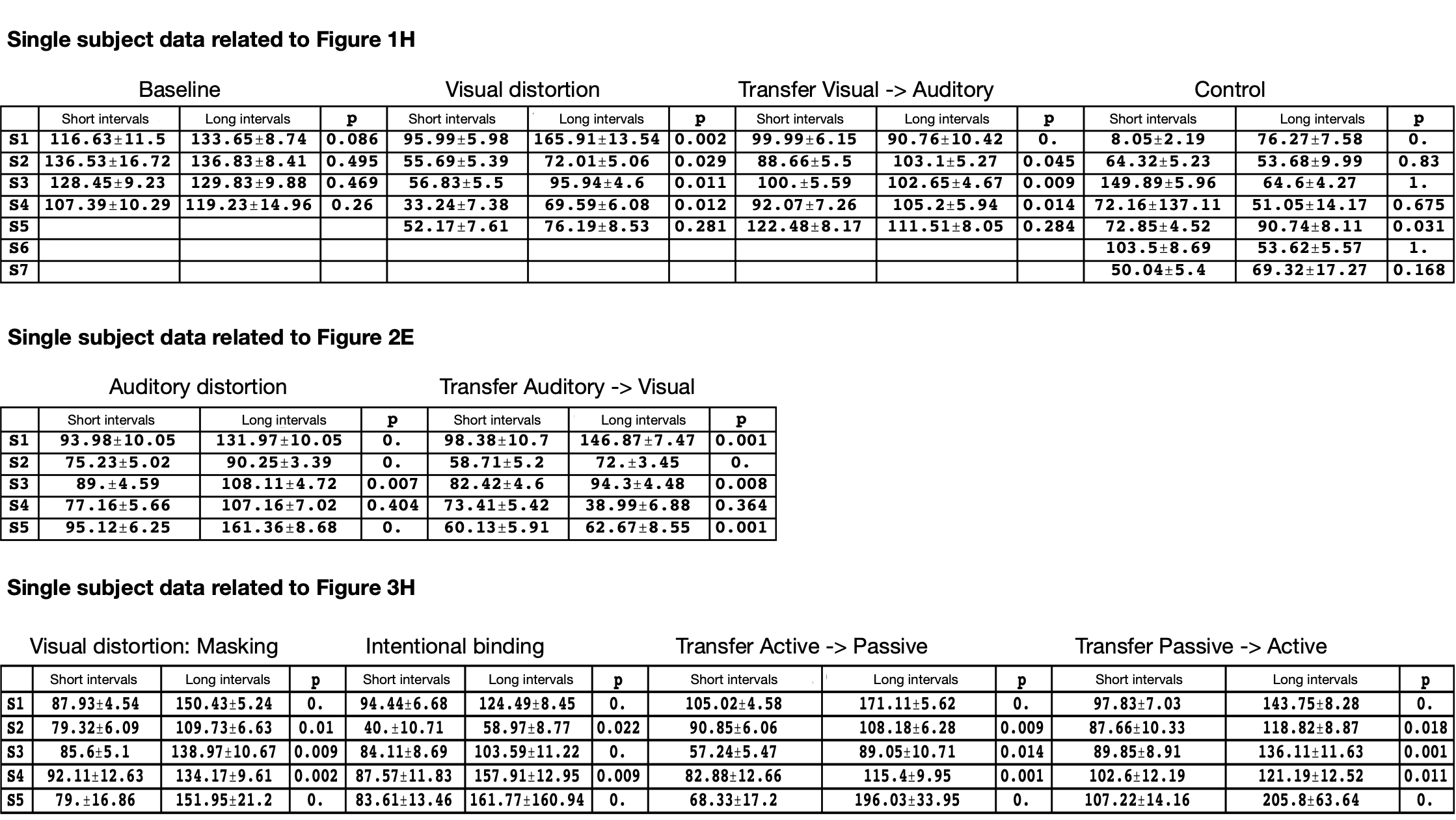


Table 1

Single subject data for interval estimations for individual observers (“int s” indicate short intervals and “int l” long intervals). Each row shows one subject (S1-S7). Data for each subject were derived by resampling the original responses and fitting a psychometric function to the resampled data. This procedure was repeated 1000 times. The data shown in the table represent the mean of the 1000 points of subjective equality with the standard error. Significance was tested with a bootstrap t-test.


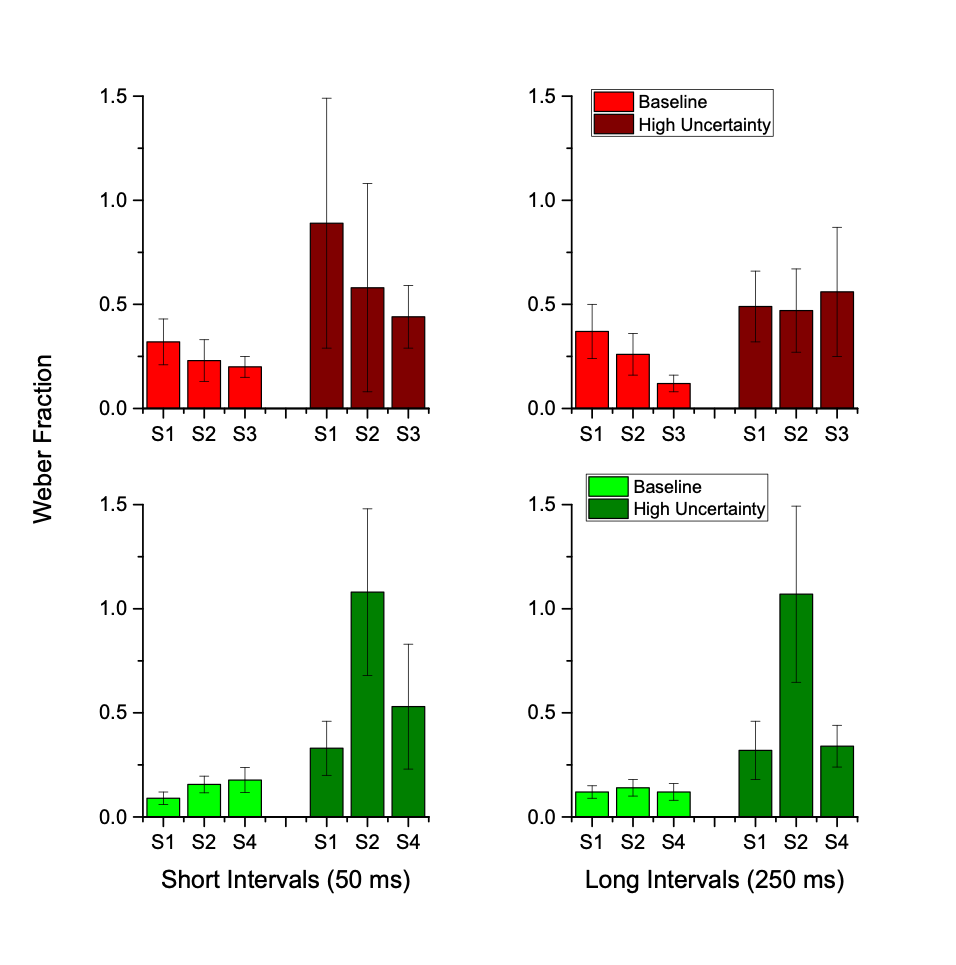


**Figure S1. Weber fractions for duration judgments either of visual stimuli (top panels) or auditory stimuli (bottom panels) in low and high uncertainty conditions.** Three subjects performed two pilot experiments in which they had to compare two intervals marked either by visual stimuli (two bars (25x3°) flashed briefly (17 ms) ±7° above or below fixation) or two brief white noise bursts. Each subject performed 3 sessions of 60 trials intermingling two durations (50 and 250 ms). Crucially stimuli could be presented either in a baseline – low uncertainty - version (left panels) or in a high uncertainty condition which putatively inferered with the reliability of the markers (right panels). In the visual condition, the high uncertainty was obtained by masking one of the two bars delimiting each interval. In the auditory condition the high uncertainty was obtained via monaural presentation of one of the two tones delimiting the interval. Standard fitting of psychometric curves was performed and Weber fraction (i.e. JND/PSE was obtained). It is clear from inspection how the high uncertainty condition (right panels) increases Weber Fractions in all subjects and conditions with all but one condition reporting a worsening of more than a factor of 2. In total 4 subjects participated. Observer 1 was one of the authors (GMC). The others were naïve to the purpose of the experiment. Error bars were S.E. assessed via bootstrap.
